# Stimulus Specific to Age-Related Audio-Visual Integration in Discrimination Tasks

**DOI:** 10.1177/2041669520978419

**Published:** 2020-12-13

**Authors:** Yanna Ren, Zhihan Xu, Sa Lu, Tao Wang, Weiping Yang

**Affiliations:** Department of Psychology, College of Humanities and Management, Guizhou University of Traditional Chinese Medicine, Guiyang, China; Department of Foreign Language, 165069 Ningbo University of Technology, Zhejiang, China; Department of Light and Chemical Engineering, 562658 Guizhou Light Industry Technical College, Guiyang, China; Department of Psychology, Faculty of Education, Hubei University, Wuhan, China

**Keywords:** audio-visual integration, discrimination task, race model, older adults

## Abstract

Age-related audio-visual integration (AVI) has been investigated extensively; however, AVI ability is either enhanced or reduced with ageing, and this matter is still controversial because of the lack of systematic investigations. To remove possible variates, 26 older adults and 26 younger adults were recruited to conduct meaningless and semantic audio-visual discrimination tasks to assess the ageing effect of AVI systematically. The results for the mean response times showed a significantly faster response to the audio-visual (AV) target than that to the auditory (A) or visual (V) target and a significantly faster response to all targets by the younger adults than that by the older adults (A, V, and AV) in all conditions. In addition, a further comparison of the differences between the probability of audio-visual cumulative distributive functions (CDFs) and race model CDFs showed delayed AVI effects and a longer time window for AVI in older adults than that in younger adults in all conditions. The AVI effect was lower in older adults than that in younger adults during simple meaningless image discrimination (63.0 ms vs. 108.8 ms), but the findings were inverse during semantic image discrimination (310.3 ms vs. 127.2 ms). In addition, there was no significant difference between older and younger adults during semantic character discrimination (98.1 ms vs. 117.2 ms). These results suggested that AVI ability was impaired in older adults, but a compensatory mechanism was established for processing sematic audio-visual stimuli.

## Introduction

Individuals are often inundated with stimuli from various sensory modalities (e.g., auditory, visual, olfactory, and somatosensory stimuli). In daily life, our brain can effectively screen and integrate effective information out of the dynamic complex information coming from the environment, thereby enabling us to acquire an appropriate perception of the outside world. The process that merges information from auditory and visual modalities is called audio-visual integration (AVI; Laurienti et al., 2006; Meredith et al., 1987; Spence, 2011; Stein & Meredith, 1993; Stein, 2012). Furthermore, studies concerning audio-visual integration have revealed that responses to audio-visual stimuli are faster and more accurate than responses to unimodal auditory or visual stimuli (Giard & Peronnet, 1999; Teder-Sälejärvi et al., 2002).

However, with ageing, the auditory threshold tends to increase, and visual acuity tends to decrease (Diederich et al., 2008; Laurienti et al., 2006), which can be attributed to the poorer health status and decline of cognitive function in older adults (Freiherr et al., 2013). Age-related audio-visual integrative studies showed an enhanced AVI effect for older adults compared with that of younger adults in auditory/visual discrimination tasks (Diederich et al., 2008; Peiffer et al., 2007; Zou et al., 2017), sound-induced flash illusion tasks (Deloss et al., 2013), semantic discrimination tasks (Diaconescu et al., 2013; Laurienti et al., 2006), and speech perception task (Sekiyama et al., 2014). These studies predicted that AVI may be a compensatory mechanism for functional decline. In contrast, the opposite results are also largely reported using the auditory/visual detection task (Mahoney et al., 2011), the auditory/visual discrimination tasks (Ren et al., 2016; Stephen et al., 2010; Wu et al., 2012), and the sentence discrimination task (Tye-Murray et al., 2010). For the aforementioned studies, simple audio-visual stimuli, semantic audio-visual stimuli, and lipreading audio-visual stimuli were employed in different studies. Compared with simple nonmeaning stimuli, much more cognitive recourse and brain regions are needed to process complex semantic stimuli (Stevenson & Wallace, 2013). In addition, the time window of AVI is an important index to evaluate when AVI occurred (Diederich et al., 2008), and Stevenson and Wallace (2013) reported an enlarged binding window for complex stimuli compared to that for simple audio-visual stimuli. Therefore, researchers have proposed that the controversial findings mainly result from the use of different experimental materials. In addition, the stimulus was present peripherally in some studies (Mahoney et al., 2011; Wu et al., 2012), while it was central in other studies (Deloss et al., 2013; Diaconescu et al., 2013; Diederich et al., 2008; Laurienti et al., 2006; Peiffer et al., 2007; Sekiyama et al., 2014; Stephen et al., 2010; Tye-Murray et al., 2010; Zou et al., 2017). There was a significant age-related decline in peripheral perceptual processing (Beurskens & Bock, 2012), so the presented location for stimuli also contributed to the conflicting results. Furthermore, the evaluation methods of AVI were also different in the aforementioned studies, such as the implementation of race model analysis (Laurienti et al., 2006; Mahoney et al., 2011; Peiffer et al., 2007; Stephen et al., 2010; Wu et al., 2012) and bimodal response enhancement/facilitation (Deloss et al., 2013; Diaconescu et al., 2013; Diederich et al., 2008; Sekiyama et al., 2014; Tye-Murray et al., 2010; Zou et al., 2017). Therefore, although numerous studies have reported age-related AVI, presently, a unified conclusion has not yet been obtained regarding how the AVI is altered with ageing. To clarify how the AVI effect is modified in the ageing brain, a systematic study was conducted with older and younger adults in the current investigation in which the responses to central simple meaningless audio-visual stimuli and semantic audio-visual stimuli were evaluated by the race model to assess the AVI effect.

In addition, most of the semantic AVI effect was investigated using alphabetic words, and logographic words were rarely used. Alphabetic language and logographic language are likely to involve both overlapping and distinct processes (McBride, 2016; Nelson et al., 2009). The AVI difference between Chinese and Finnish was investigated by Xu et al. recently, and their results indicated that the AVI was similar for Chinese and Finnish stimuli in the left superior temporal cortex but with activation specific to the Chinese stimuli observed in the left inferior frontal cortex (Xu et al., 2019). However, as we know, the ageing effect of AVI for Chinese characters has not been studied. Therefore, the ageing effect of AVI in logographic languages such as Chinese presents another intriguing question. Understanding the ageing effect of character-related integration in logographic languages may provide more insights into the entire and language-specific ageing brain. Therefore, in the current study, semantic characters were also employed as semantic materials.

To investigate the ageing effect of AVI systematically, the audio-visual discrimination task was conducted including simple meaningless images, semantic images, and semantic character stimuli. The simple meaningless visual images (ellipse with horizontal or vertical arrows) and auditory sounds (540 Hz and 560 Hz) were selected according to the study of Giard and Peronnet (1999). The semantic visual images and their corresponding sounds were selected on the basis that each animal had high naming agreement and familiarity norms for both older and younger adults (Barrett & Newell, 2015). The semantic characters (Simplified Chinese) and their corresponding flat tone speech sounds were selected according to the study by Xu et al. (2019). Here, the same experimental groups, the same task, and the same analysis method were employed by removing all the possible variates that might influence AVI to better understand the underlying mechanisms that systematically subserve audio-visual multisensory processing with ageing. Considering that AVI could occur in both the perceptual and cognitive stages, we hypothesized that the relationship of the AVI effect between older and younger adults was diverse during audio-visual discrimination in the three conditions.

## Materials and Methods

### Participants

Twenty-six healthy older adults and 26 healthy younger adults were recruited to participate in the present study, and 22 healthy older adults (60–79 years old, mean age ± *SD*, 66.90 ± 5.57) and 26 healthy younger adults (19–24 years old, mean age ± *SD*, 21.32 ± 1.22) completed the experiment successfully and were used for further analysis. All the participants were paid for their time with RMB 60 per hour. All the younger adults were college students at Hubei University, and the older adults were citizens of Wuhan City. All participants were free of neurological diseases, had normal or corrected-to-normal vision, and were naïve to the purpose of the experiment. Participants were excluded if their Mini-Mental State Examination (MMSE) scores were greater than 2.5 *SD*s from the mean for their age and education level (Bravo & Hébert, 1997). In addition, the participants who reported a history of cognitive disorder were excluded from the experiment. All participants provided written informed consent for the procedure, which was previously approved by the Ethics Committee of Hubei University and the Second Affiliated Hospital of Guizhou University of Traditional Chinese Medicine.

### Stimuli

**Figure 1.**
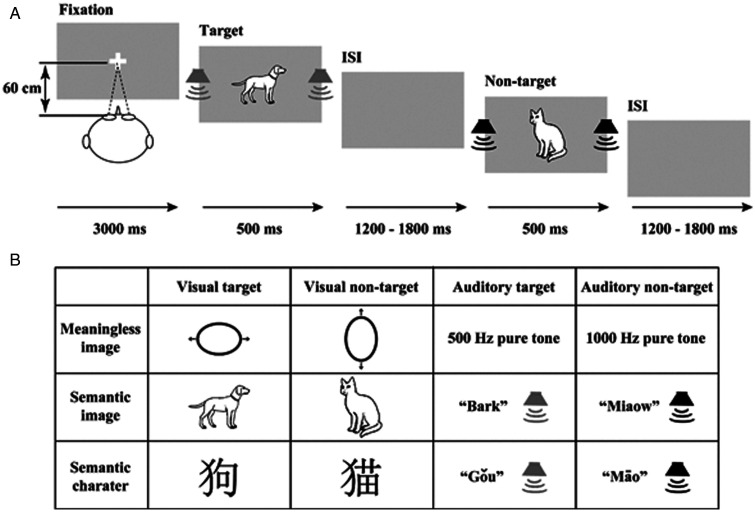
Schematic depiction of the experimental design. A: An example of a possible sequence of the audio-visual target and audio-visual nontarget stimuli in the semantic image discrimination block. B: Types of stimuli.

In the meaningless image discrimination condition, the visual target stimulus was formed by a 20% altitudinal modulation of a circle with a 5-cm diameter containing two 1-cm horizontal arrows (5.2 cm × 4.2 cm), and the auditory target stimulus was a 540-Hz sinusoidal tone. The audio-visual target was the combination of a visual target stimulus and an auditory target stimulus. The visual nontarget stimulus was formed by a 20% lateral modulation of a circle with a 5-cm diameter containing two 1-cm vertical arrows (4.2 cm × 5.2 cm), and the auditory nontarget stimulus was a 560-Hz sinusoidal tone. The audio-visual nontarget stimulus was the combination of a visual nontarget stimulus and an auditory nontarget stimulus (Figure 1B).

**Figure 2.**
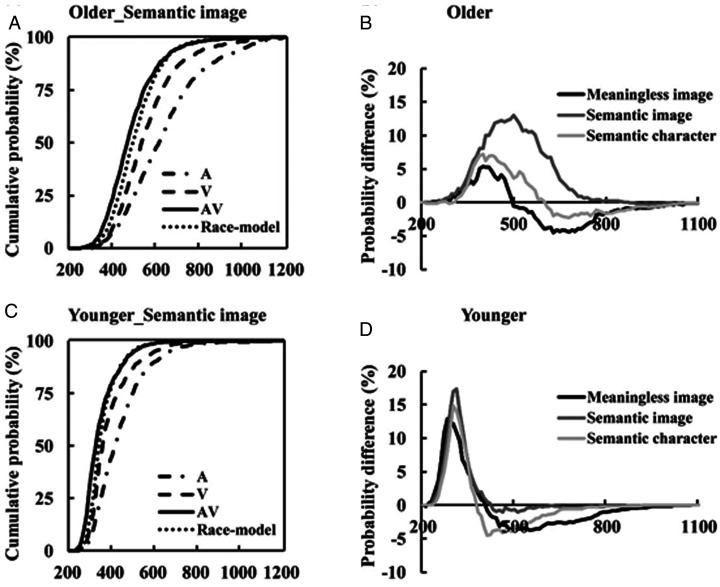
Cumulative distribution functions (CDFs) for the discrimination response times to auditory, visual, audio-visual stimuli and race model analyses in older (A) and younger (C) adults during semantic image discrimination. A higher AVI effect was found during semantic image discrimination tasks in both older (B) and younger (D) adults.

In the semantic image discrimination condition, the visual target stimulus was a black-and-white line drawing of a dog (5.2 cm × 3.8 cm) selected from Snodgrass and Vanderwart (1980), and the auditory target stimulus was the dog matched with a corresponding “bark” sound, which was downloaded from http://www.tuke88.com. The audio-visual target was the combination of a visual target dog drawing and an auditory target “bark” sound. The visual nontarget stimulus was a black-and-white line drawing of a cat (2.6 cm × 5.2 cm), and the auditory nontarget stimulus was the cat matched with a corresponding “miaow” sound. The audio-visual nontarget stimulus was the combination of a visual nontarget cat drawing and an auditory nontarget “miaow” sound (Figure 2B).

In the semantic character discrimination condition, the visual target stimulus was “狗” (5.2 cm × 5.2 cm), and the auditory target stimulus was its corresponding flat tone speech sound originating from a native male speaker of Mandarin Class A (gǒu), which was recorded using Audacity 2.3.0 (https://www.audacityteam.org/). The audio-visual target was the combination of the visual target “狗” and the auditory target “gǒu.” The visual nontarget was “猫” (5.2 cm × 5.2 cm), and the auditory nontarget was its corresponding flat tone speech sound (māo). The audio-visual nontarget was the combination of the visual nontarget “猫” and the auditory nontarget “māo” (Figure 1B).

### Procedure

The subjects were instructed to perform an audio-visual discrimination experiment, including a simple meaningless image discrimination block, semantic image discrimination block, and a semantic character discrimination block (Figure 1A), in a dimly lit and sound-attenuated room (laboratory room, Hubei University, China) with their heads positioned on a chin rest. All visual stimuli were presented on the centre of the monitor with a grey background (RGB: 192, 192, 192), and the 60-dB sound was presented through speakers located on the left and right of the monitor. At the beginning of each task, the subjects were presented with a fixation cross for 3000 ms, and then the target (A, V, AV) and nontarget (A, V, AV) stimuli were presented for 500 ms randomly with a random interstimulus interval (ISI) from 1200 ms to 1800 ms (Figure 1A). In total, 240 trials were conducted in each block with appropriate rest according to the individual’s physical condition, including 60 trials for each target stimulus type (A, V, AV) and 20 trials for each nontarget stimulus type (A, V, AV). In total, three blocks were conducted with each task lasting approximately 10 min. The order in which participants conducted the three blocks was randomized and counterbalanced across participants.

### Data Analysis

The hit rate is the percentage of correct responses (the response time falling within the average time duration ± 2.5 *SD*) relative to the total number of target stimuli. The hit rates and response times (RTs) were computed separately for each subject under each condition. Then, the data were submitted to a 2_group_ (Older, Younger) × 3_block_ (Meaningless image, Semantic image, Semantic character) × 3_stimulus type_ (A, V, AV) ANOVA (analysis of variance; Greenhouse-Geisser corrections with corrected degrees of freedom). The statistical significance level was set at *p* ≤ .05, and the effect size (*η_p_^2^*) estimates are also reported.

To evaluate the AVI effect, the race model was used to analyse the behavioural data. The independent race model is a statistical prediction model based on the cumulative distribution functions (CDFs) of the summed probabilities of the visual and auditory responses to independent unimodal visual and auditory stimuli. This model allows the direct comparison of the multisensory condition probability to the predicted probability of the unimodal conditions [P(V)+P(A)]–P(V)×P(A)] by segmenting the subject-specific CDFs for each condition using 10-ms time bins (Miller, 1982, 1986). P(V) is the probability of responding within a given timeframe in a unimodal visual trial, and P(A) is the probability of responding within a given timeframe in a unimodal auditory trial. If the probability of the response to an AV stimulus is significantly greater than that predicted by the race model (*t-test, p* ≤ .05), integration of the auditory and visual inputs is considered to have occurred. The statistical comparison between audio-visual CDFs and race model CDFs was conducted in each 10-ms bin, and the time interval for the occurrence of AVI was defined as time window of AVI (Diederich et al., 2008). The redundant nature of the bimodal audio-visual conditions was defined by subtracting a subject’s race model CDFs from his/her audio-visual CDFs in each time bin to generate a difference curve for each subject. The time spanned from the presentation of the target to the maximal benefit is defined as the peak latency, which was used to assess the time point when AVI occurred together with the time window of AVI as in our previous study (Xu et al., 2020). In addition, the positive area under the curve (AUC) was calculated to evaluate AVI ability (Ren et al., 2016).

## Results

### Hit Rates and RTs

The hit rate under all conditions was greater than 90% (Table 1). The 2_group_ (Older, Younger) × 3_block_ (Meaningless image, Semantic image, Semantic character) × 3_stimulus type_ (A, V, AV) ANOVA for hit rates revealed a significant main effect of the stimulus type, F(2, 92) = 11.830, *p* < .001, *η_p_*^2^ = 0.205, showing a higher hit rate for the audio-visual target than that for individual visual or auditory targets (AV > V > A), and no other significant main effect or interaction was observed (all *p* ≥ .160). These results indicated a facilitated response for AV stimulus, and a comparative hit rate for both older and younger adults.

**Table 1.** Mean Response Times (ms) and Hit Rate (%) With Standard Deviations (*SD*s) for the Audio-Visual Discriminations in Each Block.

	Older		Younger	
	RT (ms)	Hit rate (%)	RT (ms)	Hit rate (%)
Meaningless image				
V	555 (74)	98 (3)	383 (59)	98 (2)
A	687 (88)	96 (5)	459 (69)	97 (4)
AV	519 (73)	99 (2)	358 (50)	98 (2)
Semantic image				
V	563 (82)	97 (3)	386 (54)	98 (2)
A	636 (89)	96 (8)	435 (61)	98 (2)
AV	480 (55)	99 (2)	343 (46)	98 (2)
Semantic character				
V	554 (67)	97 (3)	381 (44)	98 (2)
A	646 (79)	99 (3)	441 (79)	98 (2)
AV	503 (61)	99 (2)	354 (50)	98 (2)

RT = response time; V = visual; A = auditory; AV = audio-visual.

The 2_group_ (Older, Younger) × 3_block_ (Meaningless image, Semantic image, Semantic character) × 3_stimulus type_ (A, V, AV) ANOVA for RTs (Table 1) revealed a significant main effect of the stimulus type, F(2, 92) = 218.880, *p* < .001, *η_p_*^2^ = 0.826, showing a faster response to the audio-visual target than that to the auditory or visual target (AV > V > A), and a main effect of group, F(1, 46) = 10.583, *p* < .001, *η_p_*^2^ = 0.688, showing a faster response to the target by the younger adults than that by the older adults. In addition, the main effect of block was also found, F(2, 92) = 4.342, *p* = .020, *η_p_*^2^ = 0.088, showing a faster response in semantic image discrimination than that in meaningless image and semantic character discriminations. In addition, interactions for Group × Stimulus, F(2, 92) = 13.444, *p* < .001, *η_p_*^2^ = 0.226 and Task × Stimulus, F(4, 184) = 11.362, *p* < .001, *η_p_*^2^ = 0.198, were also found. Further post hoc analysis showed that the response times were faster for younger adults than those for older adults (all *p* < .001), with a faster response to the audio-visual target than that to the auditory or visual target (AV > V > A, all *p* < .001), and a faster response in meaningless image discrimination tasks than that in semantic image or semantic character discrimination tasks (Meaningless image > Semantic image > Semantic character, all *p* < .001). In addition, although there was no significant difference during visual meaningless image, visual semantic image, and visual semantic character discriminations (all *p* ≥ .617), faster responses during audio-visual semantic image discrimination compared to those for audio-visual meaningless image (*p* = .006) and audio-visual semantic character (*p* = .003) discriminations, and slower responses during auditory meaningless image discriminations than those during auditory semantic image (*p* = .005) or auditory semantic character (*p* = .012) discriminations were found. No other significant interactions were observed for RTs (all *p* ≥ .155).

### Race Model Comparisons

To evaluate the diversity of the AVI effect between older and younger adults and among different audio-visual discrimination blocks, the race model was used to analyse the RTs. The AVI effect was calculated by subtracting the race model CDFs from the audio-visual CDFs in each condition, such as that for semantic image tasks for older (Figure 2A) and younger (Figure 2C) adults. A significant AVI effect was found in all audio-visual discrimination tasks (all *p* < .05, one-sample *t-test*), and the AVI effect was greater in semantic image discrimination tasks than that for meaningless image or semantic character discrimination tasks (Semantic image > Semantic character > Meaningless image) for both older (Figure 2B) and younger (Figure 2D) adults.

**Figure 3.**
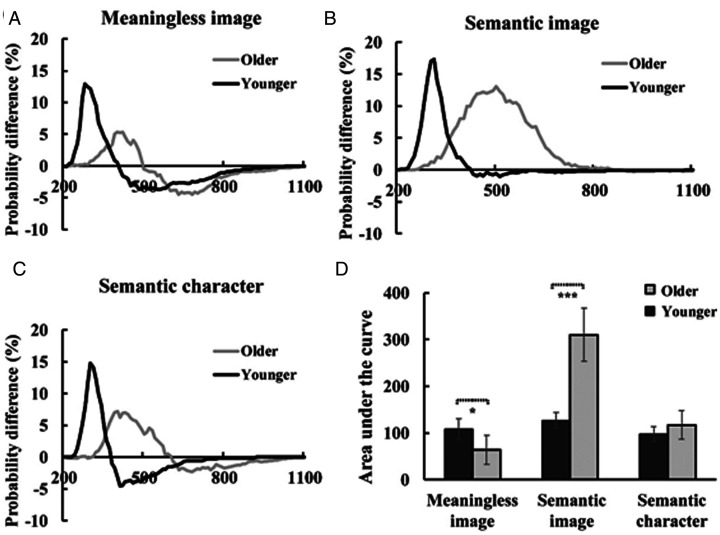
A delayed AVI effect was found during meaningless image (A), semantic image (B) and semantic character (C) discrimination tasks. In addition, significant age-related diversity of the AVI effect was found in meaningless image and semantic image discrimination tasks but not in semantic character discrimination tasks (D). Error bars indicate the SEM.

The AVI effect was lower (63.8 ms vs. 108.8 ms) and delayed (400 ms vs. 280 ms) for older adults compared to that for younger adults during meaningless image discrimination (Figure 3A, 3D, and Table 2). However, the AVI effect was higher (310.3 ms vs. 127.2 ms) and delayed (500 ms vs. 310 ms) for older adults compared to that for younger adults during semantic image discrimination (Figure 3B, 3D and Table 2). During semantic character discrimination, there was no difference in the AVI effect between older and younger adults (98.1 ms vs. 117 ms, *p* = .117), but the AVI was also significantly delayed for older compared to that for younger adults (400 ms vs. 300 ms; Figure 3C, 3D and Table 2). In addition, in the meaningless image discrimination task, the time widow for AVI was from 320 ms to 480 ms for older adults and from 220 ms to 350 ms for younger adults. In the semantic image discrimination task, the time widow for AVI was from 340 ms to 680 ms for older adults and from 240 ms to 370 ms for younger adults. In the semantic character discrimination task, the time widow for AVI was from 330 ms to 560 ms for older adults and from 260 ms to 350 ms for younger adults. These results indicated that the AVI was delayed in older adult than younger adults, and the older adults exhibited a longer time window.

**Table 2.** Area Under the Curve (AUC, ms), Peak Latency (ms), and Time Window of AVI (ms) in the Audio-Visual Discrimination in Each Block.

	Older			Younger		
	Meaningless image	Semantic image	Semantic character	Meaningless image	Semantic image	Semantic character
AUC	63.8	310.3	117.2	108.8	127.2	98.1
Peak latency	400	500	400	280	310	300
Time window	320–480	340–680	330–560	220–350	240–370	260–350

## Discussion

To systematically investigate the age-related AVI effect, an audio-visual discrimination experiment was conducted, including the use of simple meaningless images, semantic images, and semantic characters. The results illustrated that the AVI effect was lower in older adults than that in younger adults during meaningless image discrimination tasks but the results were inverse during semantic image discrimination tasks; however, there was no significant difference between older and younger adults during semantic character discrimination. In addition, the AVI was delayed, and the time window of AVI was longer for older adults than that for younger adults in all audio-visual discrimination conditions.

Mahoney et al. (2011) and Wu et al. (2012) also used simple meaningless images to investigate the age-related AVI effect; asterisks and pure tones were used for the study by Mahoney et al.’s study, and black-whiter checkerboards and white noise were used for the study by Wu et al. Their results were consistent with our present results that the AVI effect was weaker in older adults than that in younger adults. Studies conducted by Talsma’s team showed that attention influences the AVI effect in multiple stages, and the AVI effect was higher in attended conditions than that in unattended conditions (Talsma et al., 2007; 2009; 2010; Talsma & Woldorff, 2005). However, numerous behavioural and electroencephalographic studies have provided evidence for attentional deficits in older adults (Fraser & Bherer, 2013; Williams et al., 2016), which leads to less attentional resources being used to perform the cognitive task compared with the resources used by younger adults. Therefore, we assumed that the reduced AVI effect in older adults was mainly attributed to attentional decline in older adults during simple meaningless audio-visual stimuli processing. However, the AVI effect was greater in older adults than that in younger adults during semantic image discrimination, which was consistent with previous semantic audio-visual integration findings (Diaconescu et al., 2013; Laurienti et al., 2006). In the study by Diaconescu et al., MRI and MEG data were also collected, and the results showed that different from younger adults, a distinct network of posterior parietal and medial prefrontal sources was recruited in older adults when they were responding to cross-modal stimuli compared to the network recruited in response to unimodal stimuli (Diaconescu et al., 2013). Diaconescu et al. further proposed that as an adaption phenomenon, the enhanced AVI effect compensated for the uni-sensory decline. Recently, Ren et al. (2018) investigated temporal audio-visual integration in older adults and found that a significant AVI effect was elicited in a traditional visual processing brain region (the occipital cortex) in older adults but not in younger adults, which indicated that compensatory phenomena occurred to compensate for the decline in neurological function. Therefore, we proposed that older adults establish a compensatory mechanism for processing high-level cognitive semantic audio-visual stimuli, and the enhanced AVI effect was an adaptation of the ageing brain to compensate for unimodal functional decline. Furthermore, accompanying sensory process declines, reduced differentiation abilities, and regional process specificity of the ageing brain have also been extensively reported (Chong et al., 2019; Grady, 2012). Wang et al. (2018) investigated age-related functional connectivity in audio-visual temporal asynchrony integration tasks, and their results illustrated that the functional connectivity and network efficiencies of older adults revealed higher global and local efficiencies in both the theta and alpha bands. These results further suggested that higher functional connectivity between different brain regions is evoked in older adults to compensate for sensory dysfunction. In addition, Noel et al. (2016) study the audio-visual simultaneity judgment and rapid recalibration systematically across 7 to 86 years, and they found that the development and maturation for the function of perception and identification of simple stimulus was earlier than speech stimulus (Noel et al., 2016). We hypothesize that the discrimination of meaningless images mainly occurs in the low-level perceptual stage and that attention plays an important role during audio-visual integration; however, the discrimination of semantic images mainly refers to the high-level cognitive stage, and the age-related compensatory mechanism becomes active and plays an important role. During semantic image discrimination, the enhanced AVI effect was large enough to compensate for attentional decline in older adults. Therefore, it is reasonable for the AVI effect to be weaker during simple meaningless image discrimination task and higher during semantic image discrimination tasks in older adults compared with the AVI effects in younger adults.

In addition, the AVI effect was comparable for older adults with younger adults during semantic character discrimination task. Although it is commonly thought that for object naming and reading tasks, the retrieval of phonological forms is shared between semantic image and semantic character recognition, a recent study reported marked differences (Valente et al., 2016). Valente et al. recorded the time course of three distinguished phrases during the stimulus-to-response period in detail using electroencephalographic neural activity, and their results suggested similar visual processing and time courses in the two conditions for the first stage. However, compared with image naming, the common topography displayed an offset closer to response articulation in word reading, which indicated that the transition between the offset of this shared map and the onset of articulation was significantly faster in word reading (Valente et al., 2016). In the third phase, the compatible phonological processes and different temporal properties were between image naming and reading (Valente et al., 2016). This result suggested that semantic image discrimination is more complex to some degree than semantic character discrimination. In addition, resulting from individual visual and auditory dysfunction, older adults exhibit slower image naming and wording reading (De Luca et al., 2017). We hypothesize that the established compensatory mechanism is activated to some degree during semantic character discrimination compared with simple meaningless image discrimination, but the compensatory effect is weaker than that for semantic image processing.

Furthermore, the delayed AVI effect and the longer time window of AVI in older adults compared to the effect and time window of younger adults were also extensively reported in previous studies (Ren et al., 2018; Ren et al., 2016; Wang et al., 2018; Wu et al., 2012). Colonius et al. proposed a “time-window-of-integration model,” and they presumed that the integration for cross-modal information included at least two serial stages of saccadic reaction times: an early afferent stage of peripheral processing (first stage) and a compound stage of converging subprocesses (second stage; Colonius & Diederich, 2004; Diederich et al., 2008). The first stage consists of very early sensory processing, and the processing time is assumed to be independent for unimodal sensory stimuli. If the peripheral processes in the first stage all terminate within a given time interval, multisensory integration is assumed to occur in the second stage. Compared with younger adults, older adults showed a higher threshold for the perception of auditory and visual stimuli and a slower processing speed in the first stage (Liu & Yan, 2007; Spear, 1993), which led to a delay in the second stage. Therefore, the delayed AVI might be mainly due to a unimodal functional decline. Although the response was slower for older adults, they could complete all of the audio-visual discrimination tasks successfully. Therefore, we hypothesized that the longer time widow was also a compensatory phenomenon for the impaired AVI ability in older adults.

In conclusion, AVI ability was reduced in older adults when processing simple meaningless audio-visual stimuli, but the AVI ability of older adults was comparable or even greater than that of younger adults when processing sematic audio-visual stimuli. The results further suggested that AVI ability was impaired in older adults, but a compensatory mechanism was established in the ageing brain that could be activated when processing complex semantic audio-visual stimuli.
